# Association between obstructive sleep apnea and risk of Benign vocal fold lesions: A nationwide 9-year follow-up cohort study

**DOI:** 10.1097/MD.0000000000038447

**Published:** 2024-06-21

**Authors:** Yong Tae Hong, Min Gu Kang, Min Gyu Lee, Sang Woo Yeom, Jong Seung Kim

**Affiliations:** a Department of Otorhinolaryngology-Head and Neck Surgery, Jeonbuk National University Medical School, Jeonju, Republic of Korea; b Research Institute of Clinical Medicine of Jeonbuk National University – Biomedical Research Institute of Jeonbuk National University Hospital, Jeonju, Republic of Korea; c Department of Medical Informatics, Jeonbuk National University Medical School, Jeonju, Republic of Korea.

**Keywords:** Benign vocal fold lesion, obstructive sleep apnea, voice disorder

## Abstract

Since obstructive sleep apnea (OSA) affects various parts of the body, there has been little interest about the effect of OSA on voice. The objective of this study was to evaluate the risk of benign vocal fold lesions (BVFL) in OSA patients. This study used data from the National Health Insurance Service (NHIS) database. The study group was defined as the group diagnosed with OSA between 2008 and 2011. Non-OSA groups were selected based on propensity score (PS) matching. Incidence of BVFL among participants during the follow-up was analyzed. Cox proportional hazard regression analyses were performed to evaluate the association between OSA and incident BVFL. The HR value of the OSA group calculated by considering 8 variables indicates that the risk of developing BVFL is 79% higher than that of the control group. Further, among OSA patients, patients with a history of OP had a 35% lower risk of developing BVFL. The relationships between BVFL and 7 individual variables considered were as follows: For age, HR for the 40 to 59 years group was 1.20 (95%CI, 1.09–1.32). For sex, the HR in the female group was 1.22 (95%CI, 1.10–1.35). For residential areas, the HR values for “Seoul” 1.39 (95%CI, 1.23–1.59). In the high economic status group, the HR was 1.10 (95%CI, 1.01–1.21). This observational study indicated that OSA is associated with an increased incidence of BVFL. The incidence of BVFL increased with older age, female sex, and high SES.

## 1. Introduction

Obstructive sleep apnea (OSA) is characterized by recurring episodes of partial or complete upper airway obstruction while sleeping. It is estimated that 4% to 7% of adults in the general population are affected.^[1]^ Reduced neural activation and upper airway anatomic abnormalities are the primary causes of obstructive hypopneas and apneas in patients with OSA.^[2]^

OSA has a wide range of effects on the body, including the cardiovascular, neurologic, and respiratory systems. Moreover, OSA has been linked to upper airway inflammation, such as a thicker soft palate, hypertrophic tonsils, or a thickened pharynx, as well as tongue base lymphoproliferation, which can affect one voice.^[3]^ The inflammatory reaction of the upper airway, as well as the dryness of the upper airway caused by mouth breathing, can both have a deleterious impact on the vocal cord mucosa and result in a voice issue. Work environment, psychological variables, personality traits, and voice abuse have been identified as risk factors for voice disorders. Meanwhile, recent research has revealed that people with sleep apnea have poor voice quality, which might be caused by vocal fold inflammation.^[4]^ Benign vocal fold lesions (BVFL) refer to nonmalignant growths of abnormal tissue on the vocal cords. The common BVFL are vocal nodules, vocal polyps, and vocal cysts.

The objective of this study was to assess the risk of BVFL in OSA patients, which is currently unknown. This research will help us better understand the association between OSA and vocal disorders.

## 2. Methods

### 2.1. Data source

The National Health Insurance Service (NHIS) database is used in this investigation. The NHIS provides insurance claims data for 50 million subscribers in Korea, and this study was created using data from a sample of 3.5 million persons from 2008 to 2019. This information contained general medical history information (diagnosis code, hospitalization date, treatment code, and death information) as well as patient information (age, gender, residential area, economic status, and death information). (NHIS-2021-1-689) We used The Strengthening the Reporting of Observational Studies in Epidemiology Statement.

### 2.2. Study populations

The study group was made up of people who were diagnosed with OSA between 2008 and 2011, and who had a diagnostic history based on International Classification of Diseases (ICD-10) Code G473 10th Edition. Furthermore, for a more accurate analysis, the following exclusion criteria were established: Patients diagnosed with OSA between 2011 and 2019. Patients who had ICD-10 codes J381 (vocal polyp), J382 (vocal nodule), or J383 (vocal cyst) or BVFL prior to being diagnosed with OSA. The control (non-OSA) group consisted of patients who had not been diagnosed with OSA and were matched using propensity score (PS) matching. PS was estimated with age in mind to ensure that only patients with comparable tendencies to the OSA group were included and to prevent any bias from confounders.

**Figure 1.**
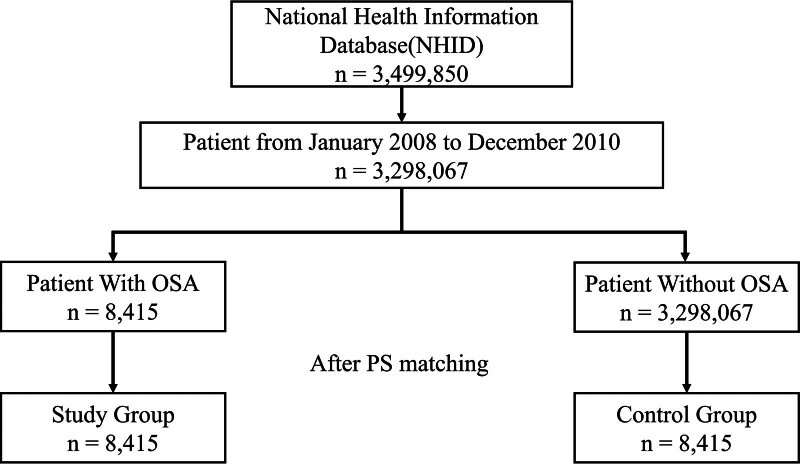
Flow chart showing the study design.

The control (non-OSA) group consisted of patients who had not been diagnosed with OSA and were matched using PS matching. PS was estimated with age, gender, economic position, and comorbidities in mind to ensure that only patients with similar tendencies to the OSA group were included and to eliminate any bias from confounders. Non-OSA groupings were then chosen using greedy method matching based on this PS. The non-OSA group had 8415 patients in total (Fig. 1).

### 2.3. Clinical variables

In all, 8 variables were created: 4 for demographics, 3 for comorbidities, and one for OSA surgery history. We separated each age group into 2 subgroups (40–60 years old, Others). Economic status was classified according to income quantiles; the top 30% had high economic status, while the bottom 70% had poor economic status. Residential areas were divided into 3 subgroups (Seoul, metropolitan, and Rural). The ICD-10 code for diagnosis and medicine prescribing was used to describe comorbidities such as hypertension (HTN), diabetes (DM), and chronic kidney disease (CKD). OSA surgical history was characterized among OSA patients as those with Q2196 (Palatopharyngoplasty), Q2197 (Uvulectomy), Q2195 (Palatopharyngoplasty < Complicated>), Q2280 (Adenoidectomy), Q2281 (Adenoidectomy < Endoscopic>), Q2300 (Tonsillectomy), or Q2310 (Lingual tonsillectomy) insurance code records prior to BVFL records.

### 2.4. Statistical analysis

The standardized mean difference (SMD) in Table 1 provides a quantitative assessment of the imbalance between the study and control groups. In general, if the SMD value is <0.1, no imbalance exists between the 2 groups. To assess the relationship between OSA and incident BVFL, Cox proportional hazard regression analysis was used. Unadjusted hazard ratios (HR) were not adjusted in Table 2, but adjusted HR were adjusted for 8 clinical factors. Further 10000-person year (PYs) is defined as the number of new disease cases in 1 year in the population at risk of disease occurrence.

**Table 1:** Characteristics of the study (OSA) and control (non-OSA) groups.

Varible	Study group	Control group	SMD
BVFL			0.127
No	7273	7613	
Yes	1142	802	
Sex			0.003
Female	1876	1886	
Male	6539	6529	
Age			0.005
Old	4992	5012	
Middle	3423	3403	
Economic status			0.003
Low	4855	4843	
High	3560	3572	
Region			0.007
Seoul	2413	2439	
Metro	1783	1781	
Rural	4219	4195	
HTN			0.013
No	5826	5875	
Yes	2589	2540	
DM			0.007
No	6621	6646	
Yes	1794	1769	
CKD			0.051
No	7879	7980	
Yes	536	435	
OP			0.942
No	5828	8415	
Yes	2587	0	

BVFL = benign vocal fold lesions, CKD = chronic kidney disease, DM = diabetes mellitus, HTN = hypertension, Metro = metropolitan, OP = operation, OSA = obstructive sleep apnea, SMD = standardized mean difference.

**Table 2:** Incidence per 1000 person-years and hazard ratios (HR) for benign vocal fold lesions during 9-yr follow-up period.

Variable	Total	Cases	10000-PYs	HR	Unadjusted HR
Total	0				
OSA					
No	8415	802	85.56	1	1
Yes	8415	1142	139.15	1.79 (1.62–1.97)	1.66 (1.52–1.82)
Sex					
Female	13068	1424	104.06	1	1
Male	3762	520	133.47	1.22 (1.10–1.35)	1.28 (1.16–1.41)
Age					
Others	10004	1026	97.61	1	1
40–59 years	6826	918	129.86	1.20 (1.09–1.32)	1.33 (1.21–1.45)
Economic Status					
Low	9698	1061	104.59	1	1
High	7132	883	118.73	1.10 (1.01–1.21)	1.13 (1.04–1.24)
Region					
Metro	3564	350	93.14	1	1
Rural	8414	912	103.32	1.08 (0.96–1.22)	1.11 (0.98–1.25)
Seoul	4852	682	136.50	1.39 (1.23–1.59)	1.46 (1.29–1.66)
HTN					
No	11701	1190	97.37	1	1
Yes	5129	754	140.69	1.06 (0.95–1.17)	1.44 (1.31–1.58)
DM					
No	13267	1305	94.03	1	1
Yes	3563	639	172.60	1.54 (1.38–1.71)	1.83 (1.66–2.01)
CKD					
No	15859	1724	103.86	1	1
Yes	971	220	224.64	1.63 (1.40–1.89)	2.15 (1.87–2.48)
OP					
No	14243	1674	111.62	1	1
Yes	2587	270	104.50	0.74 (0.65–0.85)	0.94 (0.83–1.07)

CKD = chronic kidney disease, DM = diabetes mellitus, HR = hazard ratio, HTN = hypertension, Metro = metropolitan, OP = operation, OSA = obstructive sleep apnea, PY = person-years

### 2.5. Ethical considerations

Using KNHIS-NSC data, all investigations were conducted in compliance with the Helsinki Declaration. The Institutional Review Board authorized the study (IRB file number 2022-04-021). The Institutional Review Board, which authorized the study, waived informed consent.

## 3. Results

### 3.1. Patient characteristics

This study looked at data from 8415 OSA patients and 8415 healthy controls. The male-to-female ratio in the OSA group was quite high at 77%, and the prevalence of HTN, CKD, and DM was also significant. The proportion of patients who underwent OSA surgery (OP) after being diagnosed with OSA but before the follow-up period was completed was 30.7%. Because the SMD value was <0.1 in Table 1, it can be objectively validated that the 7 variables utilized for matching were well matched. The SMD values, on the other hand, reveal that the 2 groups show differences in BVFL and OP.

### 3.2. Primary analysis

**Figure 2.**
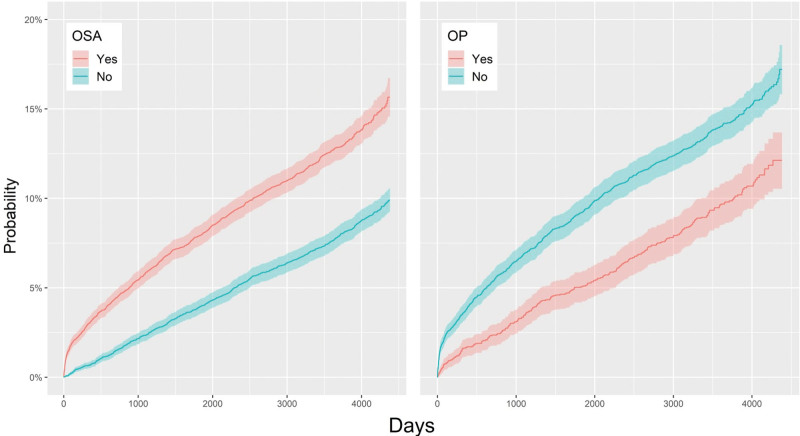
Left panel: overall cumulative hazard ratios for BVFL in the OSA group and the control (non-OSA) group. Right panel: cumulative incidence rates for BVFL in those with OSA operation and those with no OSA operation. BVFL = Benign vocal fold lesion, OSA = obstructive sleep apnea.

During the follow-up period, 1142 cases of BVFL were diagnosed among the 8415 OSA patients. The 10,000-PYs of 139.14 in Table 2 indicates that on an annual basis, there are around 139 patients per 10,000, which is 1.62 times greater than OSA patients. The OSA group has a 79% tendency to develop BVFL than the control group, according to the HR value estimated using 8 factors [HR: 1.79, 95% CI: 1.62–1.97]. In addition, individuals with a history of OP had a 35% decreased tendency to develop BVFL [HR: 0.74, 95% CI: 0.65–0.85] (Fig. 2).

### 3.3. Subgroup analyses

**Figure 3.**
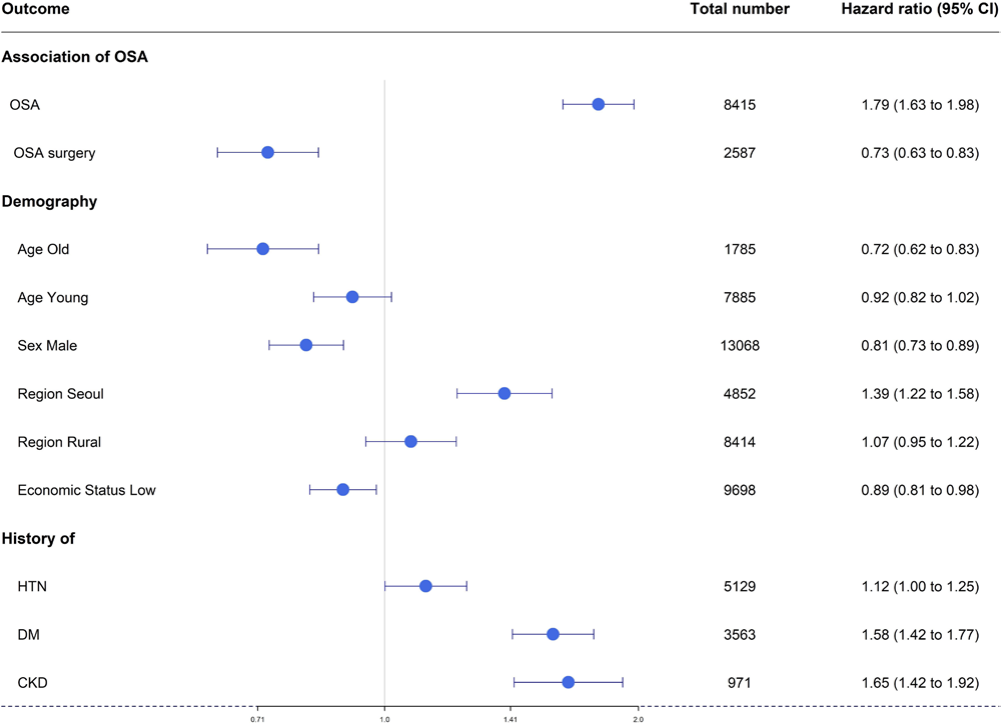
Forest plot of the cumulative hazard ratio for each factor (OSA, OSA surgery, age, sex, residential area, economic status, underlying diseases [hypertension, diabetes, chronic renal disease]).

**Figure 4.**
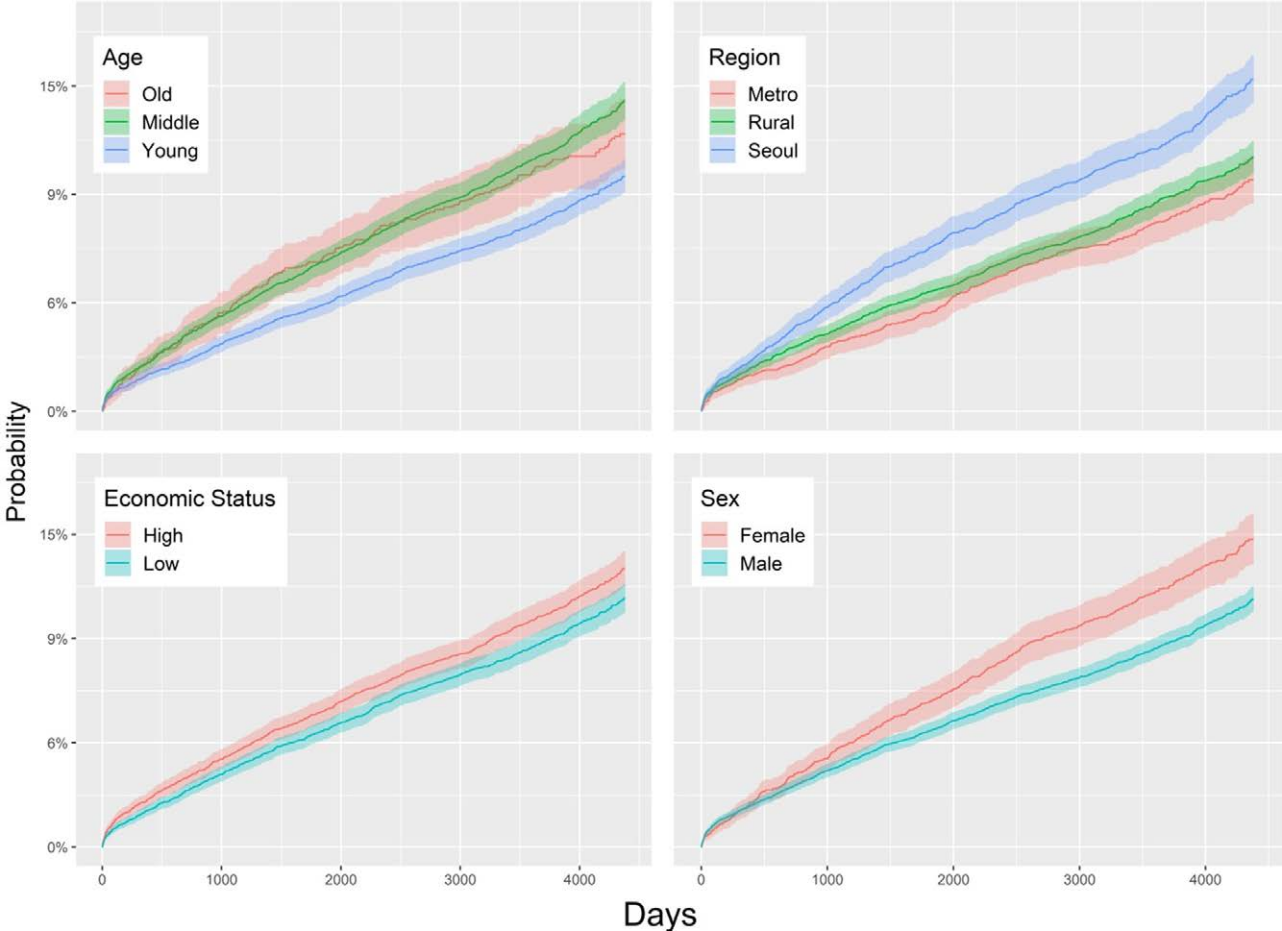
Subgroup analysis of age, residential area, economic status, and sex.

In the OSA and control group analyses, the following associations were found between BVFL and 7 individual variables: For age, HR for the 40 to 59 years group was 1.20 (95%CI, 1.09–1.32). For sex, the adjusted HR in the female group was 1.22 (95%CI, 1.10–1.35) compared to the male group. For residential areas, the HR values for “Seoul” and “Rural” were 1.39 (95%CI, 1.23–1.59) and 1.08 (95%CI, 0.96–1.22), respectively, compared to the metropolitan group. In the high economic status group, the adjusted HR was 1.10 (95%CI, 1.01–1.21) compared to the low economic status group. For underlying diseases, the adjusted HRs for the HTN, DM, and CKD groups were 1.06 (95%CI, 0.95–1.17), 1.54 (95%CI, 1.38–1.71), and 1.63 (95%CI, 1.40–1.89), respectively, compared to the equivalent groups without underlying diseases (Table 2, Figs. 3 and 4).

## 4. Discussion

The prevalence of OSA with concomitant daytime drowsiness is between 3 and 7% for adult males and 2 to 5% for adult women.^[1,3]^ OSA is defined by recurrent blockage of the upper airway during sleep, which is associated with episodic hypoxia, wakefulness, and sleep fragmentation. Upper airway anatomic anomalies, such as a thicker palate, hypertrophic tonsils, or a thickened pharynx with decreased neural activity, lead to obstructive apneas and hypopneas in OSA. OSA causes long-term damage to the cardiovascular, neurologic, and respiratory systems. According to a meta-analysis, OSA is associated with increased risks of significant adverse cardiac events, coronary heart disease, stroke, and cardiac death. OSA is also related to inflammation, since repeated bouts of hypoxia increase systemic oxidative stress and contribute to the development of a systemic inflammatory state.^[4]^ Recent research has discovered higher exhaled nitric oxide in OSA exhaled air, which contributes to upper airway inflammation.^[5]^ In this regard, OSA has been associated with the alteration of voice resonance and articulation through thickened soft palate, hypertrophic tonsils, or a thickened pharynx and tongue base lymphoproliferation.^[6]^ However, there has been little research in the effect of OSA on voice to yet.

A cross-sectional analysis of data from the national longitudinal research found a relationship between OSA and vocal disorders. People with OSA symptoms exhibited a higher prevalence of voice abnormalities than those without OSA symptoms (6.7% vs 4.7%) in a study of 14,794 young adults in the United States.^[6]^ N. Roy et al also discovered that 28% of OSA patients had vocal abnormalities, which was greater than the general population.^[7]^ However, in these research, vocal abnormalities were assessed by patients’ subjective voice pain, rather than objective voice characteristics or diseases like BVFL.

The mouth cavity, pharynx, and larynx known as the vocal tract, are structures that influence speech production. Speech impairment is caused by functional or structural abnormalities in these structures. The Bernoulli effect and enhanced pharyngeal dynamic compliance can explain pharyngeal narrowing and thickening in OSA. As a result, it is assumed that increased vocal tract compliance leads to OSA resonance and articulation problems. Many studies have observed vocal alterations following OSA surgical treatment.^[8,9]^ Eun et al, for example, found that uvulopalatopharyngoplasty alters the formant frequencies of vowels, resulting in alterations in resonance after OSA surgery.^[8]^ However, the changes that occur in the larynx in OSA patients have received less attention. Elongated epiglottis and redundancy in this structure can cause collapse and alterations in glottic and supraglottic structures.^[10]^ Furthermore, Krieger et al proposed that OSA causes repeated glottic spasms and paradoxic glottic narrowing.^[11]^

OSA patients frequently breathe via their mouths, which produces a reduction in moisture in the inhaled air, leading the vocal cords to dry. Water loss from the sol layer caused by oral breathing increased the viscosity of respiratory epithelium and overlaying mucus, increased tracheal mucus velocity, and reduced mucociliary clearance.^[12]^ Normal human oral breathing for 15 minutes revealed effects that are most likely the result of superficial dehydration on the vocal cords and increased vocal effort.^[13]^ In research of air inhalation, Hemler et al discovered that perturbation measurements were significantly higher after inhaling desiccated air than ordinary air.^[14]^ Many research, on the other hand, have observed low phonation threshold pressures in people exposed to hydrated or “wet” circumstances.^[15]^ In summary, OSA patients have long-term mouth breathing, which causes higher vocal effort due to superficial dehydration of the vocal fold.

As previously stated, there have been few reports on the impact of OSA on voice. For example, Monoson and Fox and Fox et al found the relationship between OSA and voice disorder and discovered that 60 to 70% of OSA patients exhibited a combination of phonation, articulation, and resonance abnormalities.^[16,17]^ Based on objective acoustic parameters, Wei et al recently showed that OSA patients had vibration irregularity, inadequate glottal closure, and greater hoarseness compared to normal individuals.^[18]^

Unlike prior research that investigated at voice quality, we used a nationwide cohort study to evaluate at the prevalence of BVFL in OSA patients. Multiple studies, including the National Institutes of Health epidemiology study, define voice disorder as “anytime the voice does not work, perform, or sound as it normally should, so that it interferes with communication,” implying that it is defined based on subjective symptoms based on individual judgment.^[19]^ Meanwhile, BVFLs such as vocal nodules, vocal polyps, and Reinke edema are pathologic changes in the superficial layer of the lamina propria that otolaryngologists identify with a laryngoscope. These lesions can be caused by voice abuse, misuse, smoking, alcohol consumption, or laryngopharyngeal reflux. However, the influence of OSA as a cause of BVFL has yet to be studied. In this study, the prevalence of BVFL was reported to be 1.79 times greater in the OSA group than in the control group. Those who had OSA surgery were 35% less likely to be diagnosed with BVFL during the study period.

In a subgroup analysis, the HR for BVFL was greater in female OSA patients than in male OSA patients (HR: 1.22 [1.10–1.35]). Females are more likely to have voice disorders in general, and females had a greater rate of BVFL in the literature.^[20]^ Female vocal folds contain less hyaluronic acid in the superficial layer of the lamina propria, resulting in a lower absorption ability to endure phonotrauma, according to Butler et al.^[21]^

Other risk factors for BVFL include being aged from 40 to 60 years (HR: 1.20 [1.09–1.32]), living in a major city (HR: 1.39 [1.23–1.59]), and having a higher socioeconomic status (HR: 1.10 [1.01–1.21]). Roy et al showed that among randomly chosen participants, the age range of 40 to 59 years appeared to constitute a high-risk age group for the reporting of voice problems.^[22]^ Both of these findings are similar to the findings of Russell et al, who found that teachers over the age of 50 had higher voice difficulty than younger teachers.^[23]^ Hur et al identified health inequalities among people in the United States with voice difficulties, indicating that racial minorities and those with low income tend to avoid treatment owing to a lack of transportation.^[24]^ It is well recognized in the literature that persons with poor income or insufficient health insurance face greater hurdles accessing medical services.^[25]^ Similarly, the greater prevalence of BVFL in metropolitan regions can be explained.

The adjusted HRs for the DM and CKD groups were 1.54 (95%CI, 1.38–1.71), and 1.63 (95%CI, 1.40–1.89), respectively. The laryngeal muscle is skeletal muscle which might affected by the metabolic changes induced by diabetes.^[26]^ Zaky et al reported that CKD patients were more likely to have a change of voice because dysphonia originates mainly from the weak pulmonary support of the voice.^[27]^

We used PS matching in our study to reduce selection bias and confounding variables between the 2 groups. We used PS matching with the following characteristics to achieve a fair comparison between groups: age, gender, residence, socioeconomic status, and underlying diseases. This is the first study to examine the risk of BVFL in OSA patients using large-scale real-world data. Although we produced significant results, there are some limitations that need be addressed in future study. First, despite the fact that this registry-based method resulted in a large number of unselected research participants, information about classic BVFL risk factors such as work environment, psychological factors, personality traits, voice abuse, and smoking was insufficient. Second, in our analysis, surgical treatment of OSA was related with a lower incidence of BVFL. We were unable to compare the impact of continuous positive airway pressure (CPAP), which is more often used for OSA therapy, due to a lack of data in the NHIS-NSC database, as CPAP has only recently become covered by Korean medical insurance. Third, because OSA was only recognized using diagnostic codes, the severity of the condition could not be determined.

According to this observational study, OSA is related with an increased incidence of BVFL. Subgroup analysis revealed that the incidence of BVFL in OSA patients increased with age, female sex, and high socioeconomic status. Our study also found that surgical correction of OSA decreased the incidence of BVFL. Physicians should be aware of the possibility of developing BVFL in OSA patients, which leads to poor voice outcomes.

## Author contributions

**Conceptualization:** YongTae Hong, Jong Seung Kim.

**Data curation:** YongTae Hong.

**Formal analysis:** Min Gu Kang.

**Investigation:** Min Gu Kang, Min Gyu Lee, Sang Woo Yeom.

**Methodology:** Min Gyu Lee.

**Software:** Min Gyu Lee.

**Supervision:** Sang Woo Yeom, Jong Seung Kim.

**Writing – original draft:** YongTae Hong.

**Writing – review & editing:** Sang Woo Yeom, Jong Seung Kim.
